# Metallic Nanoparticles Adsorbed at the Pore Surface of Polymers with Various Porous Morphologies: Toward Hybrid Materials Meant for Heterogeneous Supported Catalysis

**DOI:** 10.3390/polym14214706

**Published:** 2022-11-03

**Authors:** Benjamin Le Droumaguet, Romain Poupart, Mohamed Guerrouache, Benjamin Carbonnier, Daniel Grande

**Affiliations:** Univ Est Creteil, CNRS, Institut de Chimie et des Matériaux Paris-Est (ICMPE), UMR 7182, 2-8 rue Henri Dunant, 94320 Thiais, France

**Keywords:** heterogeneous supported catalysis, metallic nanoparticles, polymer, porous materials

## Abstract

Hybrid materials consisting of metallic nanoparticles (NPs) adsorbed on porous polymeric supports have been the subject of intense research for many years. Such materials indeed gain from intrinsic properties, e.g., high specific surface area, catalytic properties, porous features, etc., of both components. Rational design of such materials is fundamental regarding the functionalization of the support surface and thus the interactions required for the metallic NPs to be strongly immobilized at the pore surface. Herein are presented some significant scientific contributions to this rapidly expanding research field. This contribution will notably focus on various examples of such hybrid systems prepared from porous polymers, whatever the morphology and size of the pores. Such porous polymeric supports can display pores with sizes ranging from a few nanometers to hundreds of microns while pore morphologies, such as spherical, tubular, etc., and/or open or closed, can be obtained. These systems have allowed some catalytic molecular reactions to be successfully undertaken, such as the reduction of nitroaromatic compounds or dyes, e.g., methylene blue and Eosin Y, boronic acid-based C–C homocoupling reactions, but also cascade reactions consisting of two catalytic reactions achieved in a row.

## 1. Introduction

Metallic nanoparticles (NPs) have been the subject of intense research since their discovery. Over past decades, this research field has known a tremendous interest, mainly because of its powerful potential in diverse applications. NPs are described as objects with dimensions in the 1–100 nm range [1]. They can be prepared from inorganic [2,3] or organic matter [4], depending on the targeted applications. Metallic NPs have unique intrinsic properties that make them appealing to scientists. They possess, indeed, a great surface to volume ratio that make them suitable for applications ranging from photonics [5] to heterogeneous catalysis [6,7,8].

A catalyst, as defined by the International Union of Pure and Applied Chemistry (IUPAC), consists of a chemical substance that increases the rate of a chemical reaction, although without modifying its overall standard Gibbs energy ΔG. Thus, a catalyst is a chemical substance that it is necessary to get rid of at the end of the chemical reaction so as to recover the crude product(s), as it is regenerated during the reaction [9]. Two types of catalysts exist depending on whether or not they are in the same phase as the reactants, i.e., homo- or heterogeneous catalysts. In the last decade, a non-negligible number of scientific studies in the field have reported on catalyzed molecular reactions involving metallic NPs, either in the gas or the liquid phase. In such a case, a major drawback immediately appears to any advised chemist: how to properly, easily, and safely recover, and eventually recycle, the nanocatalyst. Indeed, finely divided metals are rather difficult to remove from the reaction mixture and recycle without processing through time-consuming and non-environmentally friendly purification processes, and they are moreover toxic, mainly because of their high volatility [10,11,12]. Some purification procedures to separate the metallic NPs from the reaction medium notably include ultracentrifugation [13], solvent evaporation [14], or ultrafiltration [15], which can be, for some of them, time-consuming and harsh techniques.

Different alternative approaches have hitherto been developed to enable an easier recovery of metallic NPs from the reaction medium. The first, and seemingly the simplest one, relies on using mixed metallic NPs composed notably of a magnetic γ-Fe_3_O_4_ core. By using a magnet at the end of the chemical reaction, the catalyst can be readily removed from the medium via a magnetic field. However, such systems tend to present issues related to their carbon-, polymer- or silica-based coatings, leading to a stability loss in harsh conditions or to a clusterization of the metallic NPs [16]. To solve these purification issues, another smarter way consists in using various matrices as supports for the adsorption of metallic nanoparticles. The supporting objects have dimensions of at least a few μm, the resulting supported heterogeneous catalysts only requiring a mere filtration to be separated from the reaction mixture [17]. Miscellaneous types of matrices have been so far considered to adsorb such metallic NPs, either inorganic (zeolites [18,19] or silica [20,21]), hybrid (especially metal–organic frameworks, MOFs [22]) or organic, namely polymeric porous materials. The porous features of some of these materials are indeed crucial as supports as they allow for development of rather high specific surface area. Polymeric materials indeed offer several undeniable advantages when compared to their inorganic or hybrid counterparts. They can be produced through low-cost chemical processes and can be functionalized in a facile and straightforward fashion, either through post-polymerization chemical modification or by suitably selecting functional monomers of interest. It is also good to notice that some of these functionalizations can be achieved in mild experimental conditions, such as by using “click” chemistry modification strategies [23]. Moreover, a variety of different synthetic strategies is already well-known to produce such porous polymeric supports [24]. Some of them have already been implemented for the preparation of metallic NPs-porous polymers, as reported elsewhere [25].

Herein, this review will focus on different strategies that have been developed so far by different research teams, but also by our group, for preparing various hybrid systems for heterogeneous supported catalysis purposes. These materials can present various pore morphologies and sizes, for instance, depending on the synthetic strategy implemented. They can be notably prepared from polymer-based electrospun mats [26], nanoporous thin films [27], bulk monolithic materials prepared by high internal phase emulsion templating [28], or phase separation or even in-capillary microsystems for flow through chemistry. Over the last two decades, several research groups have brought significant contributions to the field of hybrid materials based on porous polymers and supported metallic NPs. This review article is thus dedicated to these investigations and intends to present some significant work, which has been so far achieved, and propose some perspectives for the near future. Thus, a first section will address the diverse studies related to bulk porous and biporous polymers or to such more finely divided materials. In a second section, some interesting results regarding flow-through catalytic investigations achieved with more complex structured hybrid materials prepared in microsystems and smart nanoporous thin films prepared from diblock copolymers or membranes will be discussed.

## 2. Bulk Porous Polymeric Materials Decorated with Metallic NPs

A large variety of synthetic pathways can lead to porous polymeric materials [24]. Single-piece porous polymers, the so-called monoliths, are generally prepared from a functional monomer, a cross-linking agent, and a porogenic agent, e.g., an organic solvent. A large variety of such monoliths can be prepared depending on the nature of the functional monomer and of the cross-linker. Moreover, the pore size of those materials can be finely tuned by playing with the nature of the porogenic agent [29]. Among other porogenic agents to generate pores can be found solid particles [30,31] and linear polymers [32,33]. Alternatively, emulsion templating [34,35] and sometimes a combination of some of the above-mentioned porogens can be implemented, leading to at least two porosity levels [36,37,38,39].

### 2.1. Bulk Monolithic Supports for Metallic NPs

#### 2.1.1. Monoporous Bulk Monoliths

Budarin et al. developed the use of bio-based mesoporous polymers for catalysis applications (Table 1) [40]. Starch-based porous materials were prepared by solvent exchange between water and ethanol. Palladium (Pd) NPs were then adsorbed at the pore surface of the resulting supports. Experimentally, the porous polymer, immersed in a solution of palladium acetate, allowed for the reduction of the metal ions, thus leading to immobilization of the corresponding NPs at the pore surface. It is interesting to notice that the nanoparticle size distribution was controllable by selection of the preparation solvent. Such hybrid materials exhibited a specific surface area of 190 m^2^·g^−1^ and an average pore size of 8.2 nm through the Brunauer–Emmett–Teller (BET) as well as the Barrett–Joyner–Halenda (BJH) methods, respectively, as characterized by N_2_ physisorption. Various Pd NP-mediated and microwave-assisted C–C couplings were performed with those hybrid materials, such as the Mizoroki–Heck, Sonogashira, and Suzuki–Miyaura reactions. The authors demonstrated that microwave activation allowed for reducing the reaction time as low as 10 min while data previously reported on such catalyzed reactions, not performed under microwave irradiation, showed reaction times in the 4–12 h range. However, the benefit of such starch-based over silica-supported Pd NPs, even if expressed by the authors of the study, is very difficult to claim as no microwave-assisted catalytic reaction is reported in the literature with silica matrices.

**Figure 1:**
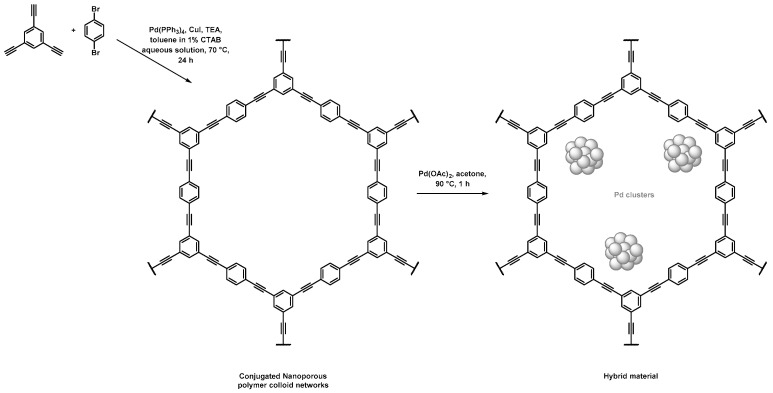
Synthetic path used for the synthesis of porous conjugated organic scaffolds by Sonogashira coupling and their use as support for the adsorption of Pd NPs [41].

Nanoporous polymeric supports were produced by Zhang et al. through a direct Sonogashira coupling between 1,3,5-triethynylbenzene and 1,4-dibromobenzene, as depicted in Figure 1 and Table 1 [41]. Thorough characterization of the porosity was investigated, notably using N_2_ sorption. The BET method gave a specific surface area of 421 m^2^·g^−1^ and a pore volume of 0.27 mL·g^−1^. For this material, calculations achieved by the nonlocal density functional theory (NLDFT), a computational quantum mechanical modelling, allowed the authors to highlight the presence of three populations of pores with sizes centered on 0.6, 1.3, and 3.1 nm. The immersion of the as-obtained porous polymers in a palladium diacetate solution in acetone under stirring at 90 °C permitted the formation of the corresponding Pd NPs. The hybrid materials permitted to achieve different Suzuki–Miyaura C–C couplings with a large variety of halogenoarenes, especially iodo- and bromo-, along with phenylboronic acid in high yields (>85%), and rather short reaction times (less than 4 h). The authors highlighted that such hybrid materials allowed for a three-fold decrease in the reaction times when compared to more conventional Pd/C catalysts (from 9 h for Pd/C to 3 h) to perform the conversion of the reactants with similar reaction yields. Those hybrid materials were recycled up to five times. No significant reduction of the catalytic activity was observed, while a leaching effect was quantified to be as low as 1%.

**Figure 2:**
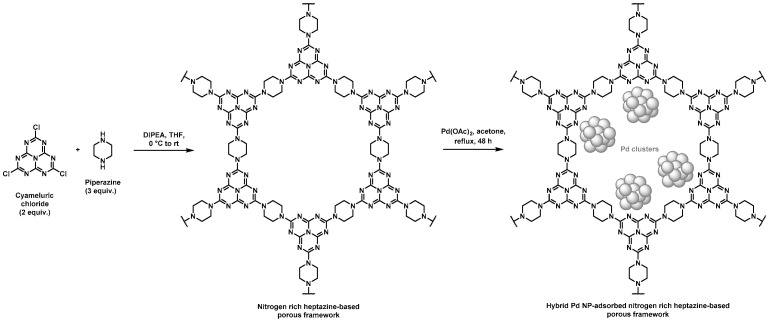
Synthetic pathway for the preparation of hybrid materials based on nitrogen rich heptazine-derived porous polymers and Pd NPs [42,43].

Nitrogen rich porous polymers have been more recently developed by Zhang et al. through a nucleophilic substitution of chlorines pending on the cyameluric chloride monomer by amines of piperazine, as shown in Figure 2 [42,43]. Such heptazine-based porous scaffolds were devoted to CO_2_ adsorption but could also find application in heterogeneous supported catalysis (Table 1). To that purpose, further adsorption of Pd NPs was performed by immersion of the resulting heptazine networks in an acetone solution containing palladium acetate under reflux, thus allowing for the self-reduction of the metal cations. Porous features of the scaffolds were determined through BET measurements using nitrogen sorption. Pore size distribution was found to be in the 2–8 nm range. Pore volumes of 0.43 and 0.33 mL·g^−1^ and surface areas of 106 and 73 m^2^·g^−1^ were found by the authors for the materials before and after immobilization of Pd NPs, respectively. These rather low values, when considering hybrid materials in which NPs should develop high surface area, were unexpected. However, Suzuki–Miyaura C–C couplings were successfully achieved with such supported catalysts in good-to-excellent yields (generally above 80%), except for the 2-bromonaphtalene/arylboronic acid and the bromobenzene/4-nitrobenzene boronic pairs, for which yields remained low (below 40%). Again, a large number of bromoarene derivative/phenylboronic acid pairs were assessed by the authors to prove the versatility of these catalytic supports. The authors claimed that the rather low yields observed for the two above-mentioned starting halogenated compound/boronic acid derivative pairs relied on the large steric hindrance of 2-bromonaphtalene as well as on the poor solubility of 4-nitrobenzene boronic acid. Recyclable character of the hybrid material was also assessed by performing five consecutive cycles. Only a limited decrease in the catalytic activity was observed and ICP measurements performed before and after five cycles only showed a slight negligible leaching phenomenon of Pd NPs.

An ingenious strategy was developed by Poupart et al. to design and prepare thiol functionalized polymeric monoliths [44]. In this work, a disulfide containing dimethacrylate-based crosslinker, i.e., bis(2-methacryloyl)oxyethyl disulfide (DSDMA), was prepared from 2-hydroxyethyl disulfide by esterification with methacryloyl chloride. The resulting crosslinker was copolymerized with ethylene glycol dimethacrylate in the presence of a porogenic solvent and a radical initiator through a photo-triggered process in a UV oven. Upon solvent removal, the observation of the porous monolith by scanning electron microscopy (SEM) highlighted the presence of an interconnected globular structure typical of the syneresis phenomenon taking place during the copolymerization in the presence of a solvent. Mercury intrusion porosimetry (MIP) confirmed this finding. Interestingly, a variation of the average pore size of the materials was observed depending on the nature of the porogenic solvent. The more polar the solvent, the larger the pore size. The presence of disulfide functions within the chemical structure of the monolith allowed for the release of thiol functions through chemical reduction in the presence of d,l-dithiothreitol (DTT). The presence of such thiol functions was highlighted by Raman spectroscopy, with the appearance of a characteristic Raman shift at 2500 cm^−1^. Finally, the presence of these thiol functions at the pore surface of the monoliths was exploited to immobilize in situ generated gold (Au) NPs through the formation of pseudo-covalent sulfur–gold bonds (Table 1). Investigations regarding the catalytic behavior of those hybrid materials was driven by following the reduction of a pollutant dye used in the textile industry, namely Eosin Y, in the presence of the hybrid material by UV-visible spectroscopy. It was also observed that the catalytic efficiency remained stable at 60% after ~10 min reaction even though a decrease was observed between the two first cycles, likely due to leakage of non-specifically adsorbed Au NPs.

#### 2.1.2. Biporous Bulk Monoliths

Biporous materials have gained some particular interest from the research community, likely due to their intrinsic properties, notably in terms of permeability, porosity, and surface area, which allowed them to be used in diverse areas, including civil engineering, tissue engineering or drug delivery. Indeed, biporous materials can benefit from each porosity level: (i) the first macroporous level offers large pores ranging from a few to hundreds of µm, providing enhanced permeability for the liquid to penetrate into the pores, but a poor specific surface area and (ii) the second level, constituted of pores having dimensions generally below 1 µm, affords a larger specific surface area, but a lower accessibility to the pores. It is good to notice that considering supported catalytic applications necessitates a high permeability regarding accessibility of the reactants to the catalytic sites, while a large specific surface area should also be envisioned to favor higher density of metal NPs on the support surface. Thus, gathering these two porosity levels in the same material may provide more efficient catalytic systems. Based on this simple consideration, material scientists are now able to design and synthesize porous polymers possessing at least two porosity levels in a precise manner, notably by independently controlling each porosity level. The preparation of biporous monoliths generally requires the combination of two porogenic agents, and different methodologies have hitherto been developed to prepare such biporous materials that encompass gas foaming, [68] temperature-induced phase separation (TIPS) [69], 3D printing [70], the double porogen templating approach, the polyHIPE technique [71], but also electrospun (co)polymer (mixture) solutions.

Electrospinning has been used up until now for the preparation of materials for environmental catalytic applications. Such electrospun materials notably allowed for the reduction of nitro-containing compounds and the treatment of hexavalent chromium (Cr^VI^) [72]. Nowadays, the implementation of such electrospun (co)polymers as catalytic supports is well-documented in the literature regarding the use of polymer mats as precursors for calcination for creating inorganic structures. On the other hand, polymer fibers possessing chelating groups, carboxylic acids, or amines, e.g., are also reported in many scientific publications of the field. Thus, Huang et al. proposed the electrospinning of a blend of polyethyleneimine (PEI) and poly(vinyl alcohol) (PVA). The resulting mats were used as supports for Au [26] and Pd [72] NPs adsorption. The corresponding Au NP- and Pd NP-based hybrid materials were successfully applied for the catalytic reduction of nitroaromatic compounds and highly carcinogenic Cr^VI^ to Cr^III^, respectively. In a similar fashion, Xiao et al. electrospun a blend of poly(acrylic acid) (PAA) and PVA. The carboxylic acid groups arising from PAA chains allowed for the chelation of sodium borohydride-mediated in situ generated Ag NPs that were successfully used for the catalytic reduction of *p*-nitrophenol [73]. Pandey et al. reported on the electrospinning of poly(ether sulfone) (PES) to prepare polymer fibers. The authors took advantage of the presence of ether sulfone moieties to initiate the growth of poly(glycidyl methacrylate) (PGMA) chains through photolysis under UV irradiation [74]. PGMA chains present at the pore surface display pending oxirane groups that were opened in the presence of hydrazine, thus allowing for the direct attachment of the reducing agents. The final step of the hybrid preparation necessitated the immersion of a palladium salt solution (PdCl_2_) with the copolymer fibers, Pd^2+^ cations self-reducing in contact with immobilized hydrazine molecules. The resulting hybrid fibers were implemented for the reduction of toxic hexavalent chromium (Cr^VI^) as well as *p*-nitrophenol but also for the less common reduction of hexavalent (U^VI^) to tetravalent uranium (U^IV^).

**Figure 3:**
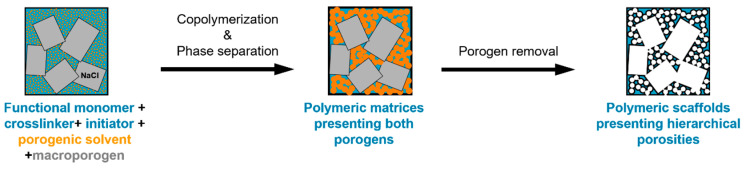
Schematic representation of the double porogen templating approach implemented for the preparation of doubly porous polymers.

**Figure 4:**
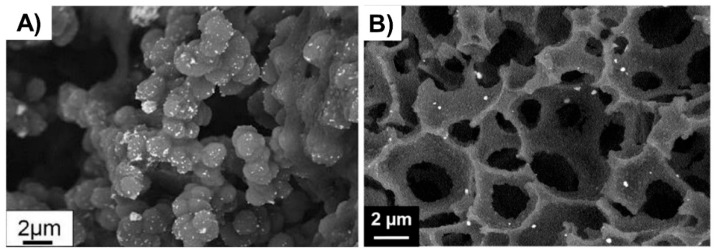
(**A**) Au NPs supported on a biporous, amino-functionalized 2-hydroxyethyl methacrylate-based monolith [46], Copyright 2017. Reproduced with permission from Elsevier Ltd.; and (**B**) Au NPs supported on a HEMA-based monolith prepared through high internal phase emulsion [48], Copyright 2018. Reproduced with permission from Elsevier Ltd.

The double porogen templating strategy, relying on the use of two distinct and independent porogenic agents, has been used notably by Ly et al. [45,46] to prepare different types of biporous polymeric monoliths, as highlighted in Figure 3. In this work, 2-hydroxyethylmethacrylate (HEMA) and ethylene glycol dimethacrylate (EGDMA) were used as the functional monomer and crosslinker, respectively, and were polymerized under UV irradiation at 365 nm in the presence of a free-radical initiator, namely AIBN. A porogenic solvent was used to generate the lower porosity level, while NaCl particles (125–200 µm) served as templates to generate the second porosity level. First, the influence of different experimental parameters was investigated regarding the porosity features of the resulting monoporous materials presenting the lower porosity level. Thus, the nature of the porogenic solvent, but also its volume ratio (with respect to the total comonomers amount), and the crosslinker to functional monomer molar ratio, were finely tuned. Thus, the more polar the solvent, the larger the pore size. Similarly, high porogenic solvent volume ratios led to larger pores and vice versa. Alternatively, poly(methyl methacrylate) beads were used to generate the macroporosity level. Upon photo-triggered copolymerization and subsequent removal of the porogenic solvent and the macroparticles, the available hydroxyl functions of the as-obtained materials were first activated with carbonyldiimidazole (CDI) and then functionalized with different amines, e.g., cysteamine, ethylenediamine, allylamine, and propargylamine. The functionalization with allylamine and propargylamine notably allowed for further modification of the pore surface through UV-mediated thiol-ene and thiol-yne “click” chemistry, respectively, using cysteamine or thioglycolic acid as model thiols. Raman spectroscopy was used as the technique of choice to follow the functionalization steps. Au NP adsorption through the in situ strategy consisted of the impregnation of the materials with an aqueous gold tetrachloroaurate (HAuCl_4_) solution, followed by the subsequent hydride-mediated reduction of Au^3+^ ions in the presence of NaBH_4_ (Table 1). Thiol-functionalized porous HEMA-based materials led to the adsorption of Au NPs with greater sizes, but more surprisingly, that also led to the highest particle leaching. On the opposite, amine functions at the pore surface of the materials led to the formation of smaller gold particles (Figure 4A) but also to better dispersed metallic NPs. This notably allowed an easier reuse of the hybrid materials for the conversion of *p*-nitrophenol into the corresponding amine in the presence of a hydride source, i.e., NaBH_4_. The catalytic reaction was very fast and easy to follow, as the solution was initially yellow due to the π→π* electron transition of the *p*-nitrophenolate ion, and it became colorless after reduction of the nitro moiety. Further, the efficiency of the biporous materials, which also have a higher specific surface area than their monoporous counterparts, was assessed. Interestingly, biporous systems showed a significantly higher catalytic efficiency than their monoporous counterparts containing either the upper porosity level or the lower porosity level. The authors claimed that this is likely due to a higher specific surface area of the doubly porous monoliths when compared to their monoporous analogues displaying the upper porosity. They also pointed out the higher accessibility of the catalyst in the doubly porous materials when compared to monoliths with only the lower porosity level. The authors finally also demonstrated that this type of hybrid catalyst can be used for the reduction of Eosin Y, paving the way toward the use of such materials for industrial wastewater depollution.

Doubly porous materials have also been prepared from the emulsion templating approach. High internal phase emulsion has been used for a long time to prepare such biporous polymeric materials in which the higher porosity level arises from the droplets of the dispersed phase and the lowest porosity level from interconnections between adjacent droplets [71]. Deleuze et al. pioneered the use of functional polyHIPEs as candidates of choice for supporting metal nanoparticle-based catalysts. In 2005, they reported on the design and synthesis of cross-linked poly(styrene-*co*-vinylbenzylchloride)-(P(S-*co*-VBC)-based [28,47] polyHIPEs as supports for in situ generated Pd NPs. Nitrogen sorption porosimetry (BET method) demonstrated that both PS and PVBC polymeric supports show a specific surface area of ca. 900 m^2^. g^−1^. At the same time, the presence of a porogenic solvent added to the HIPE polymerization feed induced a pore size distribution in the 10–80 nm range. The resulting hybrid supports, obtained by reduction of the precursor palladium salt, were used for the hydrogenation of an alkene, namely allyl alcohol [47], and for Suzuki–Miyaura cross-coupling [28] reactions (Table 1). Reaction times of 1 h and 70 h were reported for near-completion hydrogenation and coupling reactions, respectively. The authors demonstrated that PS-based catalytic supports offered good activity, even compared to commercial Pd/C, and also a satisfying reusability regarding alkene hydrogenation. Regarding the catalysis of the Suzuki–Miyaura coupling reaction, the catalytic activities of these PVBC-based hybrid materials were found to be close to those obtained with their homogeneous counterpart. Even one system showed a better activity than the well-known Pd/C powder. Finally, Suzuki–Miyaura carbon–carbon coupling reactions using these hybrid supports were successfully achieved with a wide range of substrates, demonstrating the versatility of the as-prepared materials. Some of these PS-based polyHIPEs were also implemented by the same group as supports for Au NPs [49]. To this purpose, PS-based materials were simply immerged in HAuCl_4_ solution and Au^3+^ cations were self-reduced through PS induction. Pore size in the 200–291 µm range was obtained for such materials depending on the samples, while a porosity ratio of 82% was found by mercury intrusion porosimetry. As seen in Table 1, supported Au NPs allowed for the successful and recyclable reduction of a dye, Eosin Y, under mild conditions (25 °C) within short reaction times (1 h). Recently, it was demonstrated that the synthesis of high specific surface area biporous polymers can be achieved from a reversed oil-in-water high internal phase emulsion. To this purpose, HEMA and *N*,*N’*-methylenebisacrylamide (MBA) in water and cyclohexane were emulsified in the presence of Pluronic^®^ F68 as surfactant to stabilize the concentrated emulsion [48]. The polymerization of the water continuous phase was triggered by addition of ammonium persulfate (APS) and *N*,*N*,*N’*,*N’*-tetramethylethylenediamine (TEMED). Upon polymer etching, biporous polymers presenting large pores arising from the oil droplets and smaller pores (voids) originating from the interconnections between adjacent oil droplets were obtained. A hyper-crosslinking procedure was investigated to increase the specific surface area of the polymers but also to functionalize the pore surface. Experimentally, after a two-step modification involving carbonyl diimidazole (CDI) activation followed by allylamine or propargylamine functionalization, di- and tetra-thiols were tethered to the pore surface of the biporous polymers, allowing for the hypercrosslinking of the materials, thus leading the surface to be covered with thioether moieties but also free thiols. Specific surface area, as determined by the BET method, of up to 1500 m^2^·g^−1^ could be obtained. The remaining free thiols were used to generate in situ Au NPs through impregnation with gold salts and subsequent NaBH_4_-mediated reduction (Figure 4B). Such materials were used to successfully catalyze the reduction of pollutant compounds, such as 4-nitrophenol but also Eosin Y, a dye used in the textile industry (Table 1).

### 2.2. Finely Divided Bulk Materials as Supports for NPs

Crosslinked polymeric materials are indeed successful candidates for supporting metallic NPs. However, some polymer powders can also be used. Linear polymers, if precipitated, give such functional materials. Polystyrene, being easily functionalizable through Friedel–Crafts reactions, is naturally interesting [75,76].

**Figure 5:**
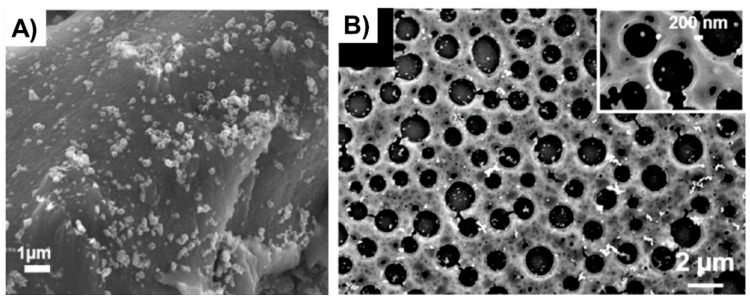
(**A**) Ag NPs supported on a powder-like PS [51], Copyright 2019. Reproduced with permission from Wiley-VCH Publishers; and (**B**) Au NPs supported on a porous PS film [55], Copyright 2017. Reproduced with permission from the American Chemical Society.

Amari et al. have described a methodology to modify linear polystyrenes (PS) and precipitate them into powder so as to support various metallic NPs. Experimentally, linear PS were submitted to nitration and subsequent reduction of the intermediate nitrocompound to afford amino-functionalized polystyrenes. Amino groups were then modified using either a chlorine-bearing triazine [50] or methyl acrylate [51]. Regarding the triazine modification, 2-aminothiazole was further added to the modified PS, so as to chelate gold ions, which were further reduced into their respective NPs through the use of a reducing chemical. The resulting Au NPs supported onto PS materials have been further used to reduce completely nitroaromatic compounds, i.e., 4-nitrophenol (93–96% reduced in 9 min for up to 5 cycles) as well as trifluralin (95% reduced in 15 min), an herbicide (Table 1). On the other hand, the acrylate-modified poly(aminostyrene), was submitted to amidation reaction in the presence of ethylenediamine. The resulting polymer has been used as a convenient support for the immobilization of silver NPs in situ generated through reduction of silver nitrate (Figure 5A). Implementation of the resulting polymer-adsorbed Ag NPs for the reduction of a pollutant dye, i.e., methylene blue, was assessed (Table 1). Such a supported catalytic reaction, monitored by UV-spectroscopy, was repeated over five consecutive cycles, with only a little decrease in the reduction yield from 97% to 91%. One should note the tendency of some metallic nanoparticles to oxidize in air, such as Ag and Cu NPs, for instance. This could lead to some loss of catalytic activity of the resulting hybrid materials, even though it was not clearly mentioned in these investigations.

**Figure 6:**
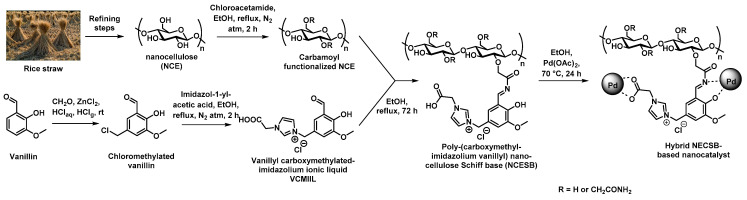
Synthetic procedure adopted for the preparation of supported Pd nanocatalysts from rice straw [52].

Recent studies from Yahya et al. reported on the implementation of a green and sustainable polymeric support prepared by refining rice straw to ionic nanocellulose Schiff base (NCESB). To this purpose, refining of the straw was processed by successive dewaxing, swelling, de-pulping, bleaching, and acidic hydrolysis steps, as shown in Figure 6 [52]. The as-obtained nanocellulose (NCE) was then treated by carbamoylmethylation to afford the resulting carbamate functionalized NCE that was modified with a vanillin derivative presenting an imidazolium ionic liquid motif to give the corresponding Schiff base-containing nanocellulose. In a final synthetic step, the hybrid material was prepared by immersion of the NCESB with a palladium acetate salt solution that allowed for the bio-reduction of the latter. TEM was used to verify the presence of well-dispersed Pd NPS presenting rather narrow pore-size distribution (5–23 nm) in the porosity of the polymer support. This green hybrid NCESB-Pd nanocatalyst was assessed regarding the Suzuki reaction using a wide range of halobenzenes and phenylboronic acid. This new catalyst exhibited amazing activity in such coupling reactions at 50 °C in short reaction times (15–60 min) and even at room temperature for longer reaction times of ca. 120 min. Finally, the recyclability of the catalyst was confirmed by monitoring the activity of the NCESB-Pd nanocatalyst on the Suzuki coupling reaction after five consecutive runs (Table 1). No significant loss of catalytic activity was observed after five cycles as 88% yield of the desired product was obtained. Different characterization techniques were used to demonstrate that the nanocatalyst was not altered in its structural and morphological nature (FTIR, XRD) while ICP-AES showed that no significant leaching (<1%) of Pd NPs was observed after these five runs, thus demonstrating the robust anchoring of the Pd NPs at the pore surface of the NCESB through carboxy, azomethine, hydroxy, and methoxy groups. The authors claimed that the new ionic nanocatalyst may pave the way for a novel generation of ionic low-cost green and highly effective nanocatalysts for organic transformation reactions.

Hybrid catalyst can also be prepared from nitrogen-based ligands [53]. Targhan et al. proposed the esterification of itaconic acid with hydroxyl-functionalized terpyridine ligand, namely 4′-(4-hydroxyphenyl)-2,2′:6′,2″-terpyridine (HPTPy), to prepare the corresponding functional monomer. The resulting bis-terpyridine functionalized ligand was then copolymerized with trimethylolpropane triacrylate (TMPTA) in the presence of MeOH/CH_3_CN (40:60) as the porogenic solvent mixture to afford the corresponding terpyridine functionalized cross-linked porous polymer. The porous morphology of the copolymer was investigated by N_2_ sorption porosimetry through the BET method. Mean pore diameter of 5 nm and surface area of 21 m^2^·g^−1^ were observed for this material. Upon refluxing a PdCl_2_ solution in EtOH with the porous polymer for 24 h, the resulting hybrid material could be obtained after purification by successive washings with EtOH. Pd NPs were characterized by XRD and TEM, notably demonstrating a disperse coverage of the material pore surface. The hybrid material was applied as a highly effective recyclable catalyst in Suzuki–Miyaura and Mizoroki–Heck coupling reactions (Table 1), allowing for high yield conversion (92 to 98%) with a large diversity of starting reagents. The reactions were thus investigated under low Pd-loading conditions and straightforward methods. The corresponding products were obtained with excellent yields (up to 98%) and high catalytic activities (TOF up to 213 h^−1^). The authors demonstrated that it is possible to separate the supported catalyst from the reaction mixture by simple centrifugation and that it could be reused for six consecutive runs with only a slight reduction in catalytic activity.

### 2.3. Nanoporous Polymer Films as Supports for NPs

**Figure 7:**
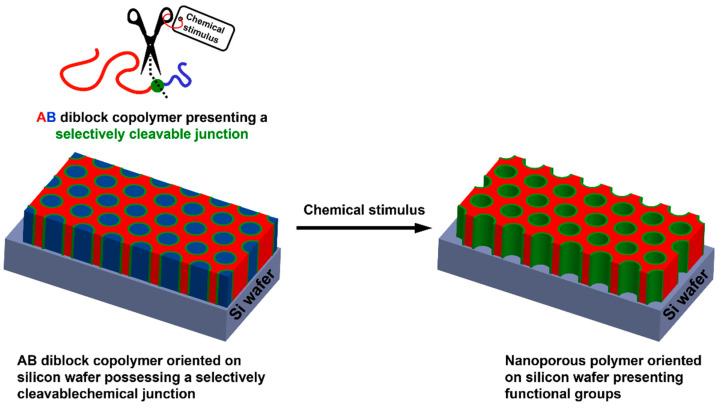
General procedure for the preparation of functional oriented nanoporous polymers from AB diblock copolymer-based thin films presenting a selectively cleavable junction.

Oriented nanoporous polymer-based thin films can also be used as interesting supports for the adsorption of metallic NPs. Thus, pore sizes of nanoporous materials are appealing for catalysis applications, as they can provide a filtration phenomenon occurring simultaneously to the catalytic activity. Such materials can be obtained from well-defined di- but also triblock copolymers containing immiscible homopolymer segments. Indeed, depending on three different parameters, i.e., the Flory–Huggins interaction parameter between both blocks (χ_AB_), *N* the number of repetition units, and *f* the volume fraction of the minority block; such AB diblock copolymers can adopt different morphologies after macroscopic orientation, including body-centered spheres, hexagonal cylinders, bicontinuous gyroids, and alternating lamellae. For instance, nanoporous polystyrene could be obtained from polystyrene-*block*-poly(d,l-lactic acid) (PS-*b*-PLA) [77,78], polystyrene-*block*-poly(ethylene oxide) (PS-*b*-PEO) [79], or polystyrene-*block*-poly(methyl methacrylate) (PS-*b*-PMMA) [80], for instance. Removing the sacrificial block could be achieved by selective chemical degradation of the minority block, but this generally required harsh experimental conditions, e.g., alkaline or acidic media, strong UV irradiation, etc. Another alternative and somehow smarter path for removing the sacrificial block lies in the selective cleavage of a chemical moiety conveniently positioned at the junction between both blocks, as depicted in Figure 7. This notably allows for using milder and more environmental-friendly experimental conditions but also positioning at the pore surface chemical functionalities of interest so as to envision further post-polymerization modification. Thus, different studies based on this synthetic strategy have emerged in the literature. Some of them notably mentioned the possibility to use photocleavable anthracene dimer-, acetal- [81], boronate [82], disulfide-, or ortho-nitro ester-based chemical junctions between the remaining and the sacrificial block [83]. This was reported by Ryu et al., who prepared oriented thiol-functionalized nanoporous thin films from a disulfide containing diblock copolymer so as to generate gold cylinders after Au NP adsorption at the pore surface [84]. Unfortunately, no application was described in this work.

Only a few of these systems have been reported in the literature describing the use of nanoporous polymers arising from diblock copolymers for heterogeneous supported catalysis. Our group has reported on the synthesis of diblock PS-*b*-PLA copolymers from a heterobifunctional initiator containing a disulfide bridge through the controlled radical polymerization of styrene via atom transfer radical polymerization (ATRP) and subsequent ring-opening polymerization (ROP) of d,l-lactide [54]. After the successful generation of both PS and PLA blocks, the resulting diblock copolymers have been submitted to a mechanical orientation through a channel die processing. The nanoporosity was generated via the selective cleavage of the disulfide bridge present between both blocks in the presence of triphenylphosphine (TPP), a chemical reducing agent. It was envisioned how such pores could be decorated with Au NPs, and it was considered the use of the resulting composite materials for supported catalysis. Thus, the advantage of the presence of thiol functions at the pore surface was taken to immobilize Au NPs through reduction of impregnated HAuCl_4_ salts. It is worth mentioning that the presence of thiol functions also allowed for grafting allyl functionalized poly(ethylene oxide) (PEO) oligomers through thermally initiated thiol-ene reaction so as to change the pore surface chemical nature, demonstrating that the wettability of the pore surface (hydrophobicity vs. hydrophilicity) could be easily tuned using such smart chemistry. Further, the Au NPs adsorbed at the pore surface of such nanoporous polystyrenes were used as supported catalysts for the reduction of 4-nitrophenol (Table 1) and showed a reduction yield of 68% after 1 h reaction. The supported heterogeneous catalysts were recycled five times in a row and did not show any loss of efficiency (reduction rates between 64 and 71%).

Acetal junction was also envisioned by our group to prepare PS-*b*-PLA diblock copolymers [55]. To that end, *p*-hydroxybenzaldehyde was first reacted with α-bromoisobutyryl bromide to give the corresponding ester intermediate that was in a second time acetalized with glycerol under acidic catalysis to afford an acetal-containing heterobifunctional initiator. PS-*b*-PLA diblock copolymers were prepared from this initiator through ATRP of styrene and successive ROP of d,l-lactide. A solution of this copolymer in THF was then spin-coated onto a silicon wafer, and the orientation of the block copolymer structure was performed via solvent vapor annealing of the resulting film. Upon removal of the sacrificial block by selective cleavage of the acetal junction in acidic conditions (trifluoroacetic acid in ethanol), an aldehyde functionalized porous polystyrene was obtained. The presence of such chemical moieties at the pore surface notably allowed for the successful functionalization through reductive amination with amines, e.g., tetraethylenepentamine (TEPA). Further, the presence of amine functions at the pore surface permitted to adsorb in situ generated Au NPs by hydride-mediated reduction of Au^3+^ cations retained at the pore surface by such chemical groups, as shown in Figure 5B. Such hybrid systems also enabled the conversion of phenyl boronic acid into biphenyl through a C–C cross-coupling reaction (Table 1). It was also implemented for the hydride-mediated reduction of *p*-nitrophenol into the corresponding amine (Table 1). More interestingly, one-pot cascade reactions consisting of the two previous successive reactions were successfully achieved with those hybrid materials. Experimentally, *m*-nitrophenylboronic acid was first submitted to C–C cross-coupling in the presence of the supported catalyst, and the 3,3′-dinitrobiphenyl was reduced when NaBH_4_ was added to the reaction mixture as the hydride source to afford as-obtained 3,3′-diaminobiphenyl. This example clearly paved the way towards the development of efficient hybrid systems for catalytic cascade reactions. Recently, another strategy based on a diblock copolymer macromolecular architecture based on the boronate ester junction [82] was developed by Bakangura et al. The convergent synthesis of such macromolecules relied on a final coupling of boronic acid- and nitrocatechol-appended polystyrene and poly(ethylene oxide) homopolymers. After spin coating of these diblock copolymers, solvent vapor casting and etching of the sacrificial PEO block in EtOH supplemented with TFA, nanoporous polymers presenting well-ordered close-packed cylindrical nanopores oriented perpendicularly to the silicon wafer supports and either catechol or boronic acid functionalities were obtained. Such nanoporous polystyrenes could be implemented for the capture of carbohydrates/diols or for the supported catalysis of molecular reactions.

## 3. Structured Hybrid Materials for Catalyzing Molecular Reactions

### 3.1. In Capillary Hybrid Systems

Flow-through chemistry has gained a tremendous interest in the last decade as it offers some undeniable advantages over more classical solution chemistry. It allows notably to process chemical reactions with a catalyst immobilized at the pore surface of (polymeric) materials prepared within flow-through systems. Such a flow chemistry has been pioneered in the second half of the 2000s [85], but a plethora of reports are now retrievable in recent literature [86,87].

**Figure 8:**
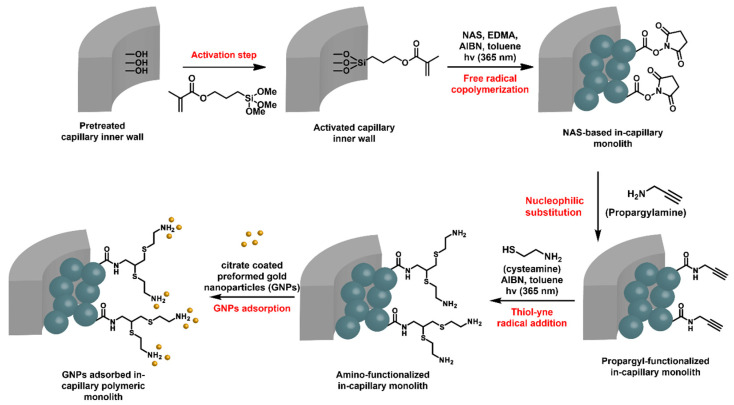
Strategy adopted for the preparation of hybrid in-capillary systems for flow through catalytic applications [88].

*N*-acryloxysuccinimide (NAS)-based monoliths were deeply investigated in-capillary as porous microsystems of choice for the adsorption of Au NPs and the further supported heterogeneous catalysis of flow-through molecular reactions [56]. The preparation of such monoliths relied again on the use of a porogenic solvent that is removed after polymerization, allowing for the generation of the pores, i.e., toluene in this particular case. The available NHS-activated ester functional group is well-known to react with nucleophiles, such as amines, for instance. In this work, the activated esters were reacted with propargylamine, as shown in Figure 8. A second UV-triggered thiol-yne radical addition with cysteamine allowed for decoration of the pore surface with amine functions. Such a functionalization step could be easily monitored by Raman spectroscopy that allowed for highlighting the disappearance of different characteristic signals of the NHS activated ester group, such as those from imide symmetric and asymmetric stretching at 1785 and 1730 cm^−1^, respectively, and from activated ester stretching at 1812 cm^−1^. In parallel, the characteristic signal from terminal alkyne was observed at about 2100 cm^−1^. The second step notably permitted spatial control of the grafting, as only the irradiated monolith was functionalized. In this way, micropatterning was possible by using photomasks. Amine functions were prone to adsorb Au NPs. A suspension of commercially available citrate stabilized Au NPs was percolated through the NAS-based amine-functionalized monolith. This example, that did not evidence flow-through catalysis, emphasized the photo-triggered strategy implemented in this study.

Since then, investigations have been expanded to this kind of in-capillary monolith and especially for the adsorption of Au NPs. In 2015, Khalil et al. developed the use of the same NAS-based monoliths as an initial platform for the grafting of ethylenediamine at the pore surface monitored by Raman spectroscopy [57]. Two distinct approaches were investigated to immobilize Au NPs. Either Au NPs were directly generated at the pore surface of monoliths by a two-step strategy involving, firstly, the percolation of a tetrachloroauric salt solution (HAuCl_4_), and secondly, the reduction of pore surface-adsorbed Au^3+^ cations by a chemical reducing agent, e.g., an aqueous NaBH_4_ solution, or colloidal gold was percolated by pumping a commercially available Au NPs suspension (20 nm in diameter) through the monolith. In the latter case, Au NPs, coated with citrate sodium salt, can be adsorbed in a robust fashion at the pore surface, as the carboxylate functions of citrate coating strongly interact with the amines at the pore surface through electrostatic interactions. Different nanogold-catalyzed reduction reactions involving nitroarenes have been investigated, notably with *o-*, *m-*, and *p*-nitrophenol as model molecules. To that extent, various experimental parameters have been investigated by the authors, such as the column length, the concentrations of the reagents, and the flow rate of the reagents solution, so as to highlight the critical parameters to take into account, so as to allow full conversion of the nitro group into the respective amine. It was notably demonstrated that in situ generated Au NPs offered a higher conversion rate than their commercially available counterparts in the same reaction conditions (flow rate, monolithic capillary length, and concentrations).

More recently, Poupart et al. also investigated the influence of the organic ligand grafted at the surface of the in-capillary monolith toward, notably, the immobilized NPs’ morphology and their dispersion at the monolith pore surface [57]. To that purpose, ethylene diamine-derived ligands were grafted at the surface of NAS-based highly permeable in-capillary monoliths. After immobilization of in situ generated Au NPs, the as-obtained hybrids, essentially differing on the grafted amine-containing ligands, were compared. Scanning electron microscopy highlighted the crucial role of the grafted amine ligand regarding the morphology, size, and surface coverage of the immobilized Au NPs at the monolith pore surface. Further, such hybrid microsystems were successfully implemented as flow-through microreactors for the catalytic reduction of nitroaromatic compounds into the corresponding amines. It was demonstrated that monolith-adsorbed Au NPs exhibited good catalytic activities in a flow-through process. This study clearly demonstrated the key role of the nature, primary vs. secondary, of the chelating amine in the morphology (shape, size, dispersion) of the supported Au NPs.

**Figure 9:**
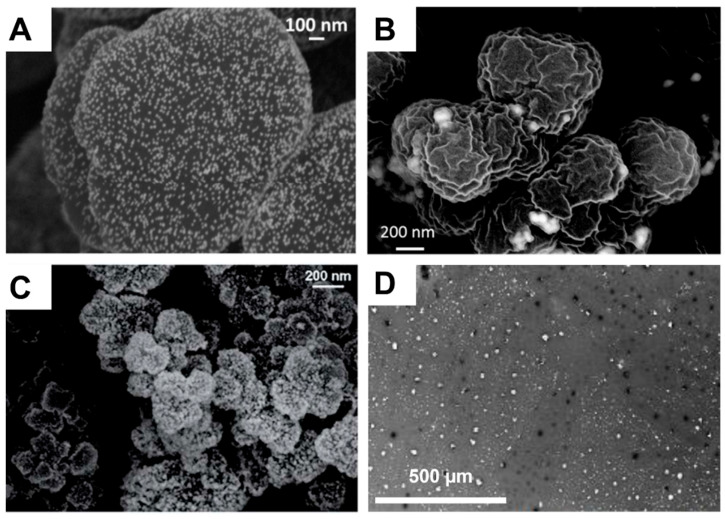
(**A**) Ex situ generated Au NPs supported on a NAS-based capillary monolith [58], Copyright 2017. Reproduced with permission from the Royal Society of Chemistry; (**B**) ex situ generated Cu NPs supported on a NAS-based monolith [59], Copyright 2015. Reproduced with permission from Elsevier Ltd.; (**C**) in situ generated Pt NPs supported on a GCMA-based monolith [60], Copyright 2016. Reproduced with permission from the Royal Society of Chemistry; and (**D**) Au NPs supported on a porous cellulose membrane [66], Copyright 2017. Reproduced with permission from Wiley-VCH Publishers.

Liu et al. also extended the scope of this strategy based on in-capillary monoliths [58]. NAS was again used as the functional monomer for their preparation. In this study, histamine, i.e., a natural compound containing both an imidazole ring and a terminal aliphatic primary amine in its structure, was grafted at the pore surface of such monoliths. The presence of the imidazole rings at the pore surface, but also its protonation in acidic conditions at pH 1, 3, or 6.5, allowed for the robust and efficient immobilization of commercially available 5-, 20-, or 100-nm Au NPs in flow-through conditions. As theoretically expected, the lower the pH of the protonation solution, the higher the gold content immobilized at the pore surface. Indeed, protonation of the imidazole rings afforded ammonium cations to create electrostatic interactions with carboxylate groups of the citrate molecules coating Au NPs. The size of the particles was also demonstrated to be a parameter to control so as to improve both the permeability of the hybrid monolith and more importantly the catalytic efficiency. All prepared samples presented a homogeneous coverage of the Au NPs at the pore surface of the histamine-grafted NAS-based monolith (see, for example, Figure 9A for 20-nm sized Au NPs). However, the 100 nm-sized Au NPs tended to aggregate and clog the interconnected porosity of the monolith, rendering the resulting microsystem inappropriate for flow-through catalytic applications. The catalytic efficiency of the as-obtained monolith-immobilized 5- or 20-nm-sized GNPs have been investigated with nitroarenes (Table 1), e.g.,4-nitrophenol, 2,5-dinitrophenol, 2,4-dinitroaniline, 2,6-dinitroaniline, and 3,5-dinitroaniline. Twenty nm-based systems showed the best catalytic efficiency. However, these results have to be taken with care, as the gold content involved was not the same when comparing 5- and 20-nm-sized NPs.

Poupart et al. demonstrated that Cu NPs could also be used as metallic NPs for nitroaromatic compound reduction (Table 1) [59]. Once again, NAS was used as the functional monomer and was polymerized in fused silica capillaries whose inner surface was preliminarily activated with 3-methacryloxypropyltrimethoxysilane (γ-MAPS). A functionalization step consisting of an amide coupling via dynamic loading of an allylamine solution through the capillary was necessary to decorate the pore surface with alkene moieties. The presence of these unsaturations allowed for the grafting of thiol-containing compounds through a UV-triggered radical thiol-ene addition, e.g., mercaptosuccinic acid. The success of this coupling reaction was successfully monitored by Raman spectroscopy. Finally, the presence of carboxylic acid functions arising from mercaptosuccinic acid favored the chelation of Cu^2+^ cations at the monolith pore surface, and their reduction by NaBH_4_ enabled them to produce supported Cu NPs. On the other hand, commercially obtained 40 to 60 nm-diameter Cu NPs suspensions were also percolated through the as-prepared microcolumns, as shown in Figure 9B, for the sake of comparison. Reduction of *o*-nitrophenol was performed on such hybrid monolithic capillaries to assess their catalytic behavior. Reduction yield of 68.5% was obtained for the preformed NPs using a flow rate of 0.3 µL·min^−1^, while lower yields were obtained using the in situ generated NPs (40 and 55% for flow rates of 4 and 1.5 µL·min^−1^, respectively).

Glycerol carbonate methacrylate (GCMA), i.e., a compound derivatized from a bio-based molecule, namely glycerol, has been used as a functional monomer for the preparation of in-capillary monoliths through free-radical copolymerization in the presence of EGDMA crosslinker and AIBN under UV-triggered radical initiation [60]. This monomer presents a carbonate-containing ring that can be then functionalized with nucleophiles, e.g., amines, through nucleophilic attack. After optimization of the reaction conditions, it has been demonstrated that these monoliths give good permeabilities when toluene and dodecanol are used as the porogenic mixture in a 40/60: *v*/*v* ratio, giving rise to pores in the 2.2 µm range but also pores with a size of around 50 nm. In situ Raman spectroscopy was used to monitor the surface modification of the monolith after percolation of allylamine. The disappearance of the carbonate ring was notably observed together with the appearance of double bonds at the pore surface of this in-capillary monolith. This notably allowed for envisioning of implementing such microsystems for both immobilization of NPs or selectors for heterogeneous supported catalysis or chromatography purposes, respectively. In this context, mercaptobutyric acid has been grafted through UV-triggered thiol-ene addition; PtCl_4_ salt aqueous solution has been then percolated through the monolithic capillary before its NaBH_4_-mediated reduction. In this way, hybrid materials were obtained within capillary microsystems and observed through SEM, as highlighted in Figure 9C. Such functional microsystems were successfully implemented for the NaBH_4_-mediated reduction of *p*-nitrophenol (Table 1), under a flowrate of 2 µL·min^−1^. It is good to notice that such allylamine functionalized in-capillary monoliths could also be modified with 1-octanethiol through thiol-ene addition and then implemented for separating a series of alkylbenzenes (namely toluene, *n*-propylbenzene, *n*-pentylbenzene and 1-phenylhexane) at a flowrate of 0.4 µL·min^−1^.

### 3.2. Hybrid Materials from Membranes

Functional polymer-based membranes can also be interesting candidates for immobilizing metallic NPs as supported catalysts. As the liquid is forced to percolate through the porosity of the membranes, such as through in-capillary microcolumns, the catalytic reaction is forced to occur. A literature survey on the subject suggested that Pd NPs present a great interest for such preformed or home-made systems [27,63,65]. The design and application in heterogeneous supported catalysis applications of diverse polymer-based membranes exhibiting nanoporosity has also been reported in the literature.

Commercially available polyethersulfone (PES) membranes having pores in the 200 nm range were notably modified by Remigny and Lahitte so as to anchor Pd NPs, the resulting hybrid membranes being implemented in several catalytic reactions, including nitrophenol reduction [61,62], hydrogenation of trans-4-phenyl-3-buten-2-one [86], or Suzuki–Miyaura cross-coupling reactions [27,62,65] (Table 1). The authors notably realized a comparison of these membranes used either in batch mode or under flow-through conditions. Flow chemistry with such membranes allowed for faster reactions. Indeed, while the reactions could be performed within a 10 s range in flow conditions, the batch mode required 6 h for full conversion. Another interesting result was that no byproducts were observed in the flow-through mode.

Other research groups mentioned the use of membranes presenting embedded NPs. Mora-Tamez et al. [66] reported on the implementation of Au NPs immobilized cellulose triacetate-based membranes. To this purpose, membranes composed of cellulose triacetate were prepared in the presence of some plasticizers (i.e., 2-nitrophenyl octyl ether and Adogen^®^ 364) and semi-interpenetrated with an inorganic phase prepared by sol-gel process in the presence of poly(dimethylsiloxane) and tetraethoxysilane. The authors claimed that the originality of the synthetic strategy arose from the extraction of Au^3+^ ions by the membranes through percolation for 5 h and their concomitant in situ reduction with a 0.01 M sodium citrate solution (Turkevitch method). The resulting hybrid membranes were characterized using SEM imaging that demonstrated the presence of Au NPs within the membranes (Figure 9D) but also by nitrogen adsorption/desorption isotherms (BET method). Specific surface area values ranging from 67 to 137 m^2^·g^−1^ and pore volume values from 0.048 to 0.097 mL·g^−1^ were obtained. Other characterizations, such as transport properties, cyclic voltammetry, and XPS, were performed to determine the gold quantity within the materials. The reduction of *p*-nitrophenol was successfully achieved through such membranes. Two types of hybrid membranes were compared in this study, one prepared from polymeric inclusion membranes (PIMs), also called AuNPs-PIM, and the other one obtained from starting polymeric nanoporous membranes (PNMs), also called AuNPs-PNM10%. The first one contains no additional porogen while the second one contains 10 wt % dimethyl phtalate as a nanoporogenic agent. The obtained results suggested a better catalytic efficiency of the AuNPs-PIM membranes, with ~95% reduction in 25 min. On the other hand, the AuNPs-PNM10% membranes reduced only ~87% of the reactant after 120 min.

Pd(0) NPs supported on a cellulose acetate membrane (CA/Pd(0)) were found to be highly efficient heterogeneous catalysts for Suzuki–Miyaura cross-coupling reactions between phenylboronic acid and a broad range of iodo-, bromo-, and electron-poor chloroarenes [67]. The synthesis of such a hybrid system started with the preparation of Pd NPs by H_2_ decomposition of Pd(acac)_2_ salt dissolved in BMI.BF4 ionic liquid at 75 °C for 1 h. Pd NPs with a diameter of 2.7 ± 0.4 nm under the form of a black suspension were obtained. Upon purification and drying, Pd NPs were mixed with a cellulose acetate solution to generate the CA/Pd(0) hybrid membrane. XRD, SEM, electron-dispersive spectroscopy (EDS), and TEM were further used to thoroughly characterize the resulting hybrid membranes. The CA/Pd(0) membrane-assisted coupling reactions were achieved under eco-friendly conditions (Table 1), i.e., phosphine-free and with water as the solvent, and gave good-to-excellent yields, depending on the nature of the haloarene counterpart nature.

## 4. Critical Appraisal

As can be seen from all the above-mentioned investigations regarding the preparation of hybrid materials consisting of metallic nanoparticles adsorbed at the pore surface of porous polymers meant for heterogeneous supported catalysis applications, different parameters are to be taken into serious consideration when designing such materials. The first one involves some inherent features of the porous materials, that is to say the porosity, the nature of the polymer, and the nature of functional groups at the pore surface. Indeed, it is well-known that the porosity should allow for efficient mass transfer across the materials to allow the reactant some easy access to the catalytic sites present on the nanometals. It has notably been demonstrated that the presence of two porosity levels in the materials allows for better catalytic efficiency [45]. The chemical nature of the functional groups present at the pore surface can have an impact on the metal chelation but also on the shape/size of the resulting nanoparticles when the in situ strategy is implemented. Many studies in this domain have favored the functionalization of the pore surface with amine groups; this notably allowed for a dense and homogeneous adsorption of metallic nanoparticles through the in situ strategy [57,58]. Other ones rely on the use of thiols or carboxylic acids. However, other chemical functions have been used and also showed some interesting results, such as thiol [44] or carboxylate functions [59], depending on the targeted metal to adsorb at the pore surface. Additionally, the chemical nature of the porous polymer has a crucial role on the morphology of the nanoparticles [89,90]. This was demonstrated by growing silver nanoparticles from glass surface-grafted polymer brushes. In that case, sphere-like shape, nanorods, and weakly branched dendritic nanostructures could be obtained as a function of the polymer chains tethered at the surface, i.e., constituted of 4-vinylpyridine, (oligo(ethylene glycol)ethyl ether methacrylate, or a mixture of both monomers. Finally, the shape of the nanoparticles has been shown to play an important role on their resulting catalytic efficiency [91]. This was notably demonstrated by comparing the catalytic performances of silver nanoparticles displaying different morphologies, namely truncated triangular, cubic, and near-spherical silver nanoparticles. The authors observed a 15-fold increase in the catalytic activity of those nanoparticles regarding the oxidation of styrene dependent of the nanoparticle shape.

## 5. Conclusions

To conclude, hybrid materials consisting of metallic NPs immobilized at the pore surface of various porous polymer-based materials have shown a strong potential in heterogeneous supported catalysis of several molecular reactions, among which are the reduction of different chemical species used in industry that are considered major pollutants. Among them, one can find nitroaromatic compounds and dyes but also metallic cations or even uranium ions. Other molecular reactions that can lead to high value-added products have also been the subject of numerous investigations from different research groups with heterogeneous supported catalysts, such as hydrogenation and C–C homo- or cross-coupling reactions. Some of these hybrid materials can even undergo one-pot cascade reactions. Different features of such nanoparticle supports are very important to take into consideration, such as the chemical functionality of the material surface, as well as the pore size and polymer morphology. On the other side, the synthetic strategy of nanoparticles is also of paramount importance as it will dictate their size and their dispersion over the porous support. More importantly, these catalytic hybrid materials can be recycled without significant loss of their catalytic efficiency. Thus, these research investigations should pave the way towards more and more efficient catalytic systems. Thus, the immobilization of organometallics or organocatalysts at the pore surface of these porous polymers may lead to the preparation of smart materials that can be used for asymmetric synthesis, for instance.

## Figures and Tables

**Table 1:** Summary of the more significant catalytic molecular reactions achieved using hybrid materials prepared from metallic NPs and porous polymeric supports.

Monomer/ Polymer Nature	Porogen Nature	Metal Nature	Metallic NP Preparation Strategy	NP Size (nm)	Catalytic Reaction Investigated	References
Starch	No porogen	Pd	in situ	4 (EtOH) and 2.5 (acetone)	Mizoroki–Heck coupling	[40]
					Sonogashira coupling	
					Suzuki–Miyaura coupling	
1,3,5-Triethynylbenzene and 1,4-dibromobenzene	No porogen	Pd	in situ	nd ^a^	Suzuki–Miyaura coupling	[41]
Heptazine and piperazine	No porogen	Pd	in situ	3−5	Suzuki–Miyaura coupling	[42,43]
DSDMA	Porogenic solvent	Au	in situ	200	Eosin Y reduction	[44]
HEMA	Porogenic solvent + NaCl particle	Au	in situ	100–150	Nitroarene reduction	[45,46]
P(S-*co*-VBC)	Emulsion templating	Pd	in situ	2, 2.5 and 2.7	Suzuki–Miyaura coupling	[28]
P(S-*co*-VBC)	Emulsion templating	Pd	in situ	12.1 to 13.8	Alkene hydrogenation	[47]
HEMA	Emulsion templating	Au	in situ	Large distribution	Nitroarene reduction	[48]
					Eosin Y reduction	
PS	Emulsion templating	Au	in situ	50–200	Eosin Y reduction	[49]
PS	Porogenic solvent	Au	in situ	7–25	Nitroarene and herbicidereduction	[50]
PS	Porogenic solvent	Ag	in situ	15–25	Methylene Blue reduction	[51]
NCESB	nd ^a^	Pd	in situ	5–23	Suzuki coupling	[52]
HPTPy	Porogenic solvent	Pd	in situ	9	Suzuki–Miyaura coupling	[53]
					Mizoroki–Heck coupling	
PS	Sacrificial block etching	Au	in situ	nd ^a^	Nitroarene reduction	[54]
PS	Sacrificial block etching	Au	in situ	100	Nitroarene reduction	[55]
					Boronic homocoupling	
NAS	Porogenic solvent	Au	in situ	nd ^a^	Nitroarene reduction	[56,57]
			ex situ	20		
NAS	Porogenic solvent	Au	ex situ	5, 20 and 100	Nitroarene reduction	[58]
NAS	Porogenic solvent	Cu	in situ	39 ± 8	Nitroarene reduction	[59]
			ex situ	68 ± 16		
GCMA	Porogenic solvent	Pt	in situ	nd ^a^	Nitroarene reduction	[60]
PES	nd ^a^	Pd	in situ	2–5, 4.7–8.4 and 6–13	Nitroarene reduction	[61,62]
				5.5 ± 1.8 and ca. 2	Alkene hydrogenation	[63,64]
				2 ± 1, 1–6, 2–5 and 6–13	Suzuki–Miyaura coupling	[27,62,65]
Cellulose triacetate	Porogenic solvent	Au	in situ	36.7 (PIMs) 2.9 (PNMs)	Nitroarene reduction	[66]
Cellulose acetate	Porogenic solvent	Pd	in situ	2.7 ± 0.4	Suzuki–Miyaura coupling	[67]

^a^ not defined in the article.

## Data Availability

Not applicable.
